# Barriers and shortcomings in access to cardiovascular management and prevention for familial hypercholesterolemia during the COVID‐19 pandemic

**DOI:** 10.1002/clc.24059

**Published:** 2023-06-01

**Authors:** Helen Huang, Keith S. K. Leung, Tulika Garg, Adele Mazzoleni, Goshen D. Miteu, Farida Zakariya, Wireko A. Awuah, Elaine T. S. Yin, Faaraea Haroon, Zarish Hussain, Narjiss Aji, Vikash Jaiswal, Gary Tse

**Affiliations:** ^1^ Royal College of Surgeons in Ireland Faculty of Medicine and Health Science Dublin Ireland; ^2^ Aston University Medical School, Faculty of Health & Life Sciences Aston University Birmingham UK; ^3^ Epidemiology Research Unit, Cardiovascular Analytics Group China‐UK Collaboration Hong Kong China; ^4^ Government Medical College and Hospital Chandigarh Chandigarh India; ^5^ Barts and The London School of Medicine and Dentistry London UK; ^6^ School of Biosciences, Biotechnology University of Nottingham Nottingham UK; ^7^ Department of Biochemistry Caleb University Lagos Lagos Nigeria; ^8^ Department of Pharmaceutical Sciences Ahmadu Bello University Zaria Nigeria; ^9^ Department of Medicine Sumy State University Sumy Ukraine; ^10^ Zhejiang University School of Medicine Hangzhou China; ^11^ Health Services Academy Islamabad Pakistan; ^12^ Royal College of Surgeons in Ireland Medical University of Bahrain Busaiteen Bahrain; ^13^ Faculty of Medicine and Pharmacy of Rabat Mohammed V University Rabat Morocco; ^14^ Department of Cardiology Research Larkin Community Hospital South Miami Florida USA; ^15^ Tianjin Key Laboratory of Ionic‐Molecular Function of Cardiovascular Disease, Department of Cardiology, Tianjin Institute of Cardiology Second Hospital of Tianjin Medical University Tianjin China; ^16^ Kent and Medway Medical School Canterbury UK

**Keywords:** atherosclerotic cardiovascular disease, cholesterol, COVID‐19, familial hypercholesterolemia, genetics, mortality

## Abstract

Familial hypercholesterolemia (FH) is a hereditary condition caused by mutations in the lipid pathway. The goal in managing FH is to reduce circulating low‐density lipoprotein cholesterol and, therefore, reduce the risk of developing atherosclerotic cardiovascular disease (ASCVD). Because FH patients were considered high risk groups due to an increased susceptible for contracting COVID‐19 infection, we hypothesized whether the effects of the pandemic hindered access to cardiovascular care. In this review, we conducted a literature search in databases Pubmed/Medline and ScienceDirect. We included a comprehensive analysis of findings from articles in English related and summarized the effects of the pandemic on cardiovascular care through direct and indirect effects. During the COVID‐19 pandemic, FH patients presented with worse outcomes and prognosis, especially those that have suffered from early ASCVD. This caused avoidance in seeking care due to fear of transmission. The pandemic severely impacted consultations with lipidologists and cardiologists, causing a decline in lipid profile evaluations. Low socioeconomic communities and ethnic minorities were hit the hardest with job displacements and lacked healthcare coverage respectively, leading to treatment nonadherence. Lock‐down restrictions promoted sedentary lifestyles and intake of fatty meals, but it is unclear whether these factors attenuated cardiovascular risk in FH. To prevent early atherogenesis in FH patients, universal screening programs, telemedicine, and lifestyle interventions are important recommendations that could improve outcomes in FH patients. However, the need to research in depth on the disproportionate impact within different subgroups should be the forefront of FH research.

## Graphical abstract

Multifaceted factors influencing atherosclerotic cardiovascular disease in familial hypercholesterolemia during the COVID‐19 pandemic.

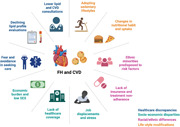

## 1 INTRODUCTION

Hereditary familial hypercholesterolemia (FH), a rare hereditary condition that causes early atherosclerosis, is characterized by extremely high levels of low‐density lipoprotein cholesterol (LDL‐C) in the bloodstream.^1^ In order for FH patients to survive, aggressive LDL‐C reduction is essential to slow the development of atherosclerosis to minimize the incidence of major cardiovascular (CV) events.^2^ Homozygous FH, a more severe form, affects approximately one out of every million individuals, while heterozygous FH is estimated to affect one in 300−500 people^3^; these statistics suggest that about 10 million people across the globe have FH.^4^ The phenotype of FH can vary across ancestries as a result of variable penetrance and expression of genetic mutations causing low‐density lipoprotein receptor (LDL‐R) defects; for example, FH could potentially occur as a result of pathogenic somatic mutations in the liver or through vertical transmission due to germinal mosaicism.^5^, ^6^ The founder effect phenomenon has been used to explain how different populations could have different causes of FH. Africans, Canadians, Lebanese, and Finns have high rates of specific mutations that make FH particularly common in these groups, with high prevalence in the United States. De Ferranti et al. reported prevalence to be between age 60−69 with obese patients more vulnerable to adverse coronary outcomes.^7^ As a result, the goal of preventative management is to reduce plasma LDL levels below 5 mmol/L.^8^

One of the main concerns regarding patients suffering from FH to contract COVID‐19, is that FH was shown to predispose the vascular lining to infectious and immune attacks, thus largely increasing the possibility to develop atherosclerotic cardiovascular disease (ASCVD).^9^ This inevitably increases the need to employ continuous and long‐term pharmacological intervention, with frequent follow‐ups and screening for ASCVD risk. However, this paradigm became obsolete during the COVID‐19 pandemic due to its direct and indirect effects on FH care.^10^, ^11^ The COVID‐19 pandemic has generated significant burdens in health management worldwide, with systems struggling to meet the needs of most individuals with comorbidities.^12^ The long‐term effects of COVID‐19 symptoms and its detrimental impact on CV care pose a major burden in healthcare systems worldwide. The impact of the COVID‐19 pandemic has been discussed in multiple conditions needing continuous long‐term management, ranging from bariatric surgery patients to individuals with acute myocardial infarction, arrhythmias, and cardiac arrest.^13^, ^14^, ^15^ Nevertheless, it is paramount to disseminate the impact of the pandemic on CV prevention in FH patients to improve future risk‐stratification protocols and raise awareness amongst clinicians. With the emerging public health concern of FH patients, this review aims to highlight the direct and indirect impact of the COVID‐19 pandemic on CV outcomes in FH. By identifying information gaps in the literature, possible recommendations can be formulated for better intervention and access to CV care.

## 2 THE GENETICS OF FAMILIAL HYPERCHOLESTEROLEMIA

**Figure 1:**
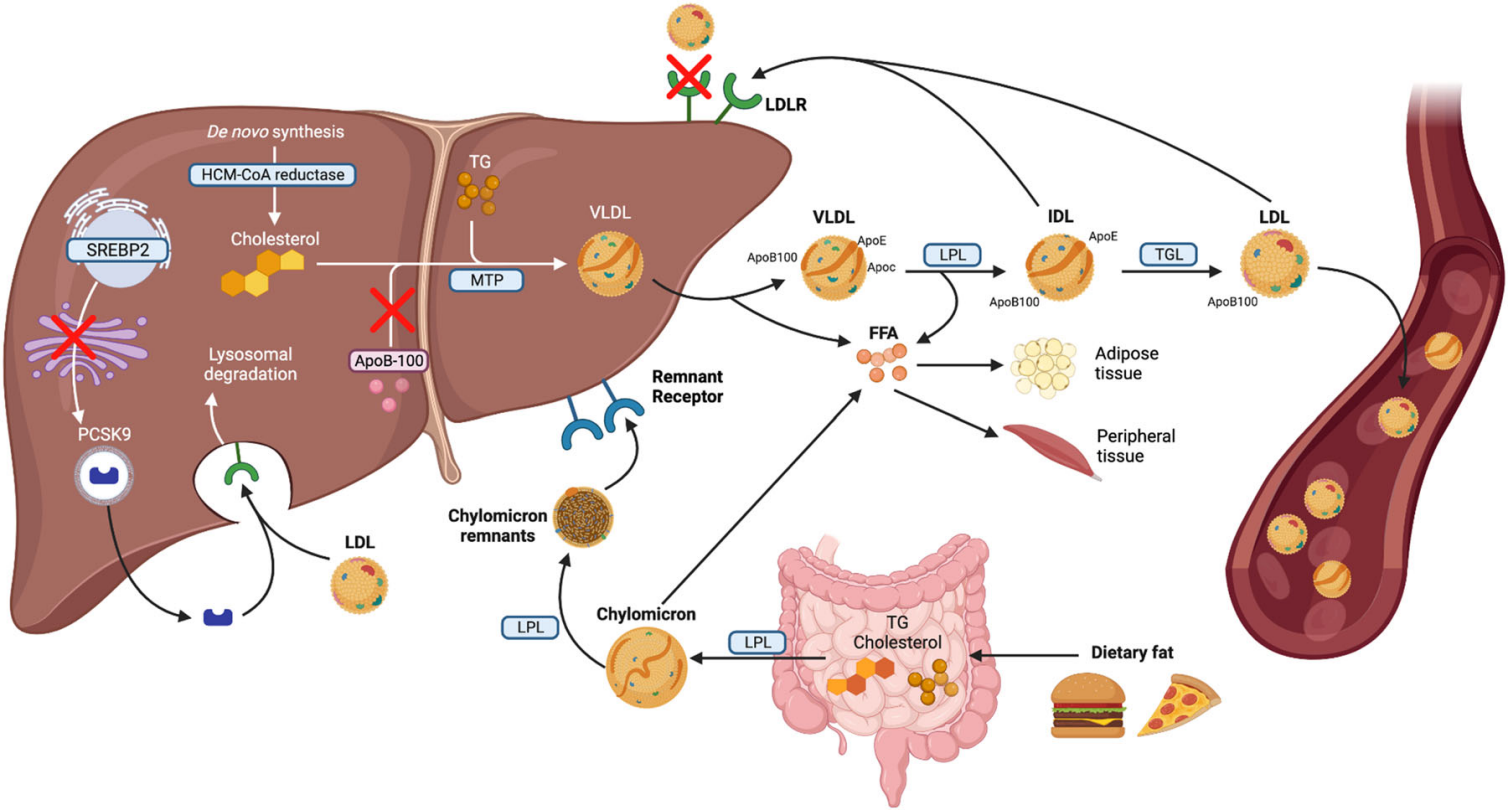
Pathways involved in lipoprotein/chylomicron synthesis and mutation sites implicated in familial hypercholesterolemia. ApoB‐100, apolipoprotein B 100; ApoC, apolipoprotein C; ApoE, apolipoprotein E; FFA, free fatty acid; HDL, high‐density lipoprotein; IDL, intermediate‐density lipoprotein; LDL, low‐density lipoprotein; LPL, lipoprotein lipase; MTP, microsomal transfer protein; PCSK9, proprotein convertase subtilisin/kexin type 9; SREBP2, sterol regulatory element‐binding protein 2; TG, triglycerides; TGL, triglyceride lipase; VLDL, very low‐density lipoprotein.

Inherited genetic mutations on chromosome 9 and subsequent enzymatic defects can explain the pathophysiology and causative mechanisms of FH in the LDL‐R, such as proprotein convertase subtilisin/Kexin type 9 (PCSK9) and apolipoprotein B^8^ (Figure 1). Clinical studies have accounted for over 1600 genetic defects and mutations of the LDL‐R in 90% of patients diagnosed with FH.^1^ When such genes are defeated by the uptake of LDL particles, cholesterol levels are also increased, leading to FH. The mechanism of action that leads to FH involves the impairment of the LDL‐R synthesis, defects in transport from endoplasmic reticulum to Golgi bodies, binding with LDL, LDL internalization, recycling of LDL‐R, defect in the apoB‐100 ligand on the LDL and gain‐of‐function mutations causing increased activity of *PCSK9*.^16^, ^17^ These lead to increased degradation of the LDL‐R which reduces LDL clearance from plasma, thereby causing an elevated level of LDL in the serum.^17^

Cellular enzymes/inhibitors for example PCSK9 secreted by the liver is vital for degrading LDL‐R and inhibiting LDL‐R recycling to the cell membrane by binding to it extracellularly to reduce cholesterol levels^18^ (Figure 1). Patients with one copy of the defective gene will experience moderate accumulation of plasma LDL while two copies of the defective gene or coexisting mutations will be more extensive due to the lack of LDL removal from plasma.^19^ As a result, this could accelerate the onset of CVD, given patients are likely to experience atherosclerotic heart disease at an early age. On the contrary, it is also important to note that mutations that inactivate PCSK9 can cause lower plasma LDL levels and can reduce coronary heart disease (CHD).^20^, ^21^ Morbidity following FH begins from a young age after defective genes have been inherited. If early diagnosis and treatment is not implemented, this condition will progress to coronary episodes and can occur between 42 and 64 years or earlier in heterozygous FH patients.^22^, ^23^ As a result, it is crucial to carry on CV screening throughout life‐time to minimize long‐term adverse outcomes.^24^

## 3 DIAGNOSTIC CRITERIAS AND GENETIC TESTING PROTOCOLS FOR FH

Several diagnostic criterias exist for establishing a diagnosis of FH, as summarized in Table 1. There are three that are widely accepted among the scientific community,^25^ including the Dutch Lipid Clinic Network (DLCN) criteria (Supporting Information: Table S1), Simon Broome criteria (Supporting Information: Table S2), and US MEDPED criteria (Supporting Information: Table S3), noting that there is not one being universally adopted as the best so far.^26^ Others include National Lipid Association expert considerations^24^ (Supporting Information: Table S4), Japan Atherosclerosis Society criteria^27^ (Supporting Information: Table S5), Welsh FH genotype scoring criteria^28^ (Supporting Information: Table S6), which is a modified version of DLCN criteria, and FAMCAT criteria^29^ (Supporting Information: Table S7).

**Table 1:** Comparison of FH diagnostic criterias.

Criteria	DLCN	SB	MEDPED	NLA	JAS	Wale	FAMCAT
Family history of premature CAD	+	+		+	+	+	+
Family history of tendon xanthomas	+	+				+	
Family history of hypercholesterolemia	+	+	+	+	+	+	+
Patient premature CAD	+			+		+	
Patient premature PVD	+					+	
Presence of tendon xanthomas	+	+		+	+	+	
Presence of Corneal arcus	+			+		+	
Elevated LDL‐C	+	+	+	+	+	+	+
Elevated triglycerides						+	+
Genetic mutation	+	+		+			
Gender							+
Presence of diabetes							+
Presence of CKD							+

*Note*: Reproduced and modified with permission, from McGowan et al.^25^

Abbreviations: DLCN, Dutch Lipid Clinical Network; FAMCAT, FAMilial hypercholesterolemia case ascertainment identification tool; FH, familial hypercholesterolemia; JAS, Japanese Atherosclerotic Society; LDL‐C, low‐density lipoprotein cholesterol; MEDPED, make early diagnosis to prevent early death; NLA, National Lipid Association; SB, Simon Broome.

The use of genetic testing to diagnose FH varies globally, with countries like Netherlands and Norway which offer testing for all suspected FH cases, to countries like Russia and those in Asia or Africa where genetic testing is not widely performed.^30^, ^31^ A positive pathogenic mutation provides a definitive diagnosis of FH; however, a negative test should never exclude suspicion of FH. Three commonly tested genes including *LDL‐R*, *ApoB*, *PCSK9* are tested, with a targeted panel sequencing of these genes yielding a positive result in 70%−80% of patients with definitive FH.^32^ Whole exome or genome sequencing (WES/WGS) is an alternative or second‐line method for those with negative panel sequencing results.^33^ Evidence toward the use of WES/WGS is still controversial, as there are a lack of novel causative genes that have been strongly identified, with exception to novel mutations in the aforementioned genes.^34^, ^35^, ^36^ Although the novel finding of *STAP1* involvement in FH is promising, its clinical significance and utility in FH yet to be established.^37^

## 4 PREEXISTING CV COMORBIDITIES IN FH INCREASE THE RISK OF COVID‐19 INFECTION AND HOSPITALIZATION

Patients who already have ASCVD seem to be more likely to contract COVID‐19 and have more severe disease with poorer clinical outcomes.^38^ Therefore, there may be a markedly increased risk for and severity of COVID‐19 infection in people with FH, particularly those with HoFH, as well as a tendency to develop ASCVD episodes.^39^ The entry of the SARS‐CoV‐2 virus into the host cell via the ACE2 receptor may also be increased in the presence of elevated cholesterol levels, as cholesterol‐rich regions of the viral membrane were hypothesized to accelerate spike‐mediated cell−cell fusion.^40^ Therefore, if they contracted COVID‐19, individuals with HoFH may be much more at risk of adverse events than individuals with HeFH. The prognosis of these participants could be morbid, as available studies suggest that a cytokine storm perpetuated by the COVID‐19 infection could destabilize atherosclerotic plaque, increasing the risk of suffering a myocardial infarction.^41^ Despite long‐term cholesterol‐lowering medication, HoFH is likewise characterized by a systemic inflammatory phenotype, and the much higher Lp(a) levels reported in HoFH participants (as in HeFH) may increase their risk for atherothrombotic events following viral infection.^42^

**Figure 2:**
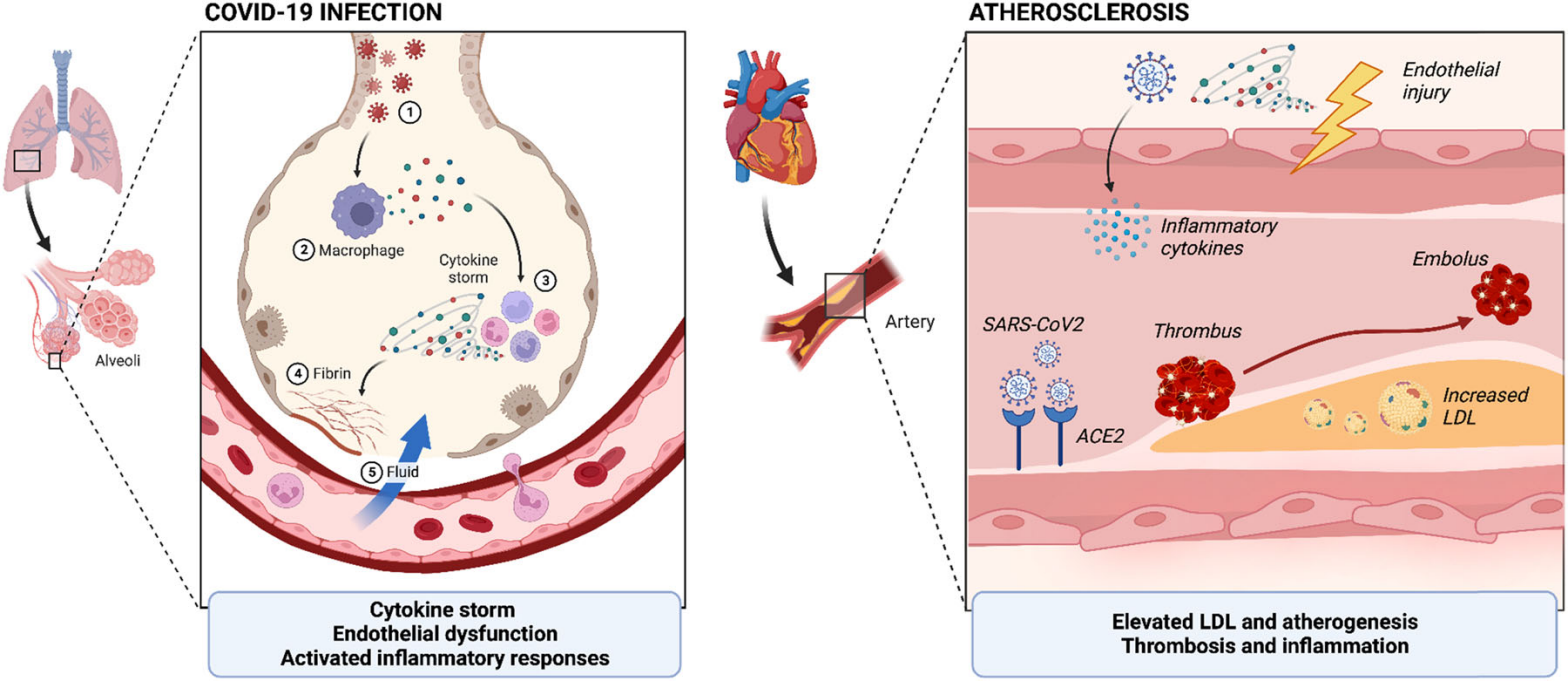
Two‐hit hypothesis of COVID‐19 infection and preexisting atherosclerosis in familial hypercholesterolemia. ACE2, angiotensin convertase enzyme 2; LDL, low‐density lipoprotein.

In the acute phase of the infection, FH patients with COVID‐19 appear to be at a high risk for COVID‐19 consequences, and over the long term, they are likely to experience accelerated atherogenesis^39^ (Figure 2). Both HeFH and, in particular, HoFH individuals are expected to have hypercholesterolemia‐induced endothelial dysfunction from birth because increased levels of LDL‐C are already present prenatally and the degree of dysfunction correlates with serum LDL‐C level.^40^ Additionally, many FH patients have increased serum Lp(a), an endothelium‐damaging lipoprotein variant that poses a significant risk factor for the development of atherosclerosis. Whilst acute coronary syndromes in people with severe COVID‐19 seem to be caused by thrombus formation in the epicardial coronary arteries during the acute stage of the disease, dysfunction of the myocardial microvascular endothelium, the coronary microcirculation, and seems to progress during the convalescent and chronic stages of the illness.^43^, ^44^ Inflammation and thrombosis from COVID‐19 infection can both interact to drive CV risk, which often occur through the surge of pro‐coagulation and proinflammatory activity^39^ (Figure 2). In addition to this, COVID‐19 may cause a continuous acceleration of atherosclerosis overtime. Because FH patients previously carry elevations of LDL‐C from birth, a “two‐hit” mechanism may explain why the risk of an acute sequelae of COVID‐19 infection is attenuated in these patient demographics.^45^ The additional damage from viral infection and subsequent activated inflammatory responses in COVID‐19 infection can further damage the endothelium in HeFH and HoFH patients. As a result, COVID‐19 infection may cause a continuous acceleration of atherosclerosis overtime if the root cause of viral vulnerability is dependent on lipid levels. Moreover, recent literature signifies that FH patients are at a predisposed risk to developing thromboembolisms, which may also be significantly higher given that venous thromboembolism was a commonly reported risk associated with COVID‐19 infection.^46^ Therefore, it is recommended that statins ought to be given during the acute, convalescent, and chronic stages of COVID‐19 for patients with FH. The beneficial outcomes of statins have been widely discussed in the literature, and was found to reduce the risk of all‐cause mortality in severe COVID‐19 infection while also reducing the risk of venous thromboembolism.^47^ After complete recovery from the COVID‐19 infection, it should be urged to continue taking a statin at effective doses.

## 5 EXACERBATING FACTORS OF THE COVID‐19 PANDEMIC THAT NEGATIVELY INFLUENCED CV MANAGEMENT AND OUTCOMES OF FAMILIAL HYPERCHOLESTEROLEMIA

### 5.1 Healthcare discrepancies and COVID‐19‐related fear preventing effective screening

The burden of FH has significantly increased over the past decade, with a 10, 20, and 23‐fold higher prevalence in subjects with IHD, premature IHD, and severe hypercholesterolemia, respectively.^48^ The sudden emergence of COVID‐19 called for all FH patients to be considered as having a high risk for cardiac complications,^49^ which in turn increases the predisposition of concomitant COVID‐19‐associated atherothrombotic complications and mortality.^50^, ^51^ As international healthcare resources needed to be redistributed thereby limiting the resources usage for noncommunicable disease.^52^ This, along with fear of infection, has led to a decrease in medical care and subsequent hospital admission of patients with acute coronary syndrome according to various studies.^53^, ^54^

The sudden burden on healthcare services due to the COVID‐19 pandemic led to many deterred hospital visits, doctor−patient interactions, screening, diagnosing, and preventive efforts due to social distancing. The resources allotted to cardiology, after the reorganization of the healthcare system, were drastically reduced and were largely limited to managing cardiac emergencies, especially, critical cardiac presentations in patients with COVID‐19.^55^ The number of lipidologist consultations were reportedly lower than before the lockdown (33.5% vs. 100.0%, *p* < .001), with a decline in lipid profile evaluations (56.5% vs. 100.0%, *p* < .01).^11^ This decline can be attributed to the widely observed pause in active screening and diagnosing of FP patients. Unidentified FH cases, owing to the lack of screening, were perhaps at a greater risk of experiencing CVD complications. As a result of reduced consultations, teleconsultation substitutions, and postponement of active LDL‐C measurements, diagnosed FH cases were poorly managed and also a culprit of increased CVD risk.^55^

The fear of COVID‐19 infection was an important topic of discussion within the medical community, as the decline in consultations may also be attributed due to fear of patients contracting COVID‐19 infection.^56^ It was reported that 33.3% of patients avoided seeking medical care during this time,^57^ which may well explain the decline in proportion of FH patients willing to undertake lipid profile analysis, vascular imaging evaluations, and doctor consultations. The main factor contributing to these results, which were obtained from FH patients via telephone survey, was the fear of SARS‐CoV‐2 transmission. This result was consistent with two earlier findings that assessed how SARS‐CoV‐2 infection affected the healthcare system. In fact, 65% of respondents in the Italian EPICOVID19 web‐based survey reported fear of SARS‐CoV‐2 contagion for both themselves and their family members.^58^ In comparison to pre‐COVID‐19 statistics, there are fewer patients with serious heart attacks being admitted to hospitals—on average, a 50% decline—according to most of the doctors and nurses who responded to the European Society of Cardiology (ESC) poll. Moreover, 48% of patients who did visit the hospital did so after the ideal window for urgent care had passed. An untreated heart attack due to patient reluctance will further life‐threatening complications and this outweighs the mortality associated with COVID‐19.^11^

### 5.2 Socioeconomic disparities and financial insecurity perpetuated by COVID‐19 pandemic

Socioeconomic status influences the kind of lifestyle that people can adopt and the quality of cholesterol‐lowering drugs they can purchase to manage their conditions. As a result, low‐socioeconomic status and issues with health literacy are major limiting factors in accessing nonpharmacological care, such as lifestyle modifications to control comorbidities and often are presented with fewer options. Low SES was linked with an increased incidence of dyslipidemia due to the established literature indicating a higher propensity of unhealthy dietary behaviors imposed by limited financial freedom.^59^, ^60^ Financial security is often an influential component for baseline testing, as prior screening is significantly disproportionate in these demographics.^61^

FH patients require specialist input for long‐term management of CV risk which can impact financially disadvantaged individuals.^62^ This remains a serious barrier to proper prevention, given the higher costs needed to treat FH, especially during the pandemic. The economic burden of these patient cohorts was quantified in a recent systematic review, wherein FH or nonfamilial dyslipidemia faced significant economic burdens from CVD, with an annual average expenditure ranging from $17 to $259 million in these communities alone.^63^ Often, these costs disproportionately affected FH patients who had previous CV events, where the significant economic burden was attributed by healthcare utilization, such as in‐patient services and interventions during hospitalizations, as well as following up with secondary prevention for FH.^63^ This was supported by a burden model developed in Turkey, which determined that the indirect economic burden associated with FH with CVD accounting for 67.5% of overall costs.^64^

The COVID‐19 pandemic, which saw the lockdown of activities worldwide, led to a deterioration in CV care, especially in those with underlying disease and worsened the inequality in financial security, employment, housing, and diet of people with low socioeconomic status.^65^, ^66^ Patients with FH are already at risk of reduced work productivity due to the complexity of the condition totaling to a loss of about AUD 101 366 per susceptible individual in a lifetime.^67^ With the emergence of pandemic‐related public health orders, there was a decline in the workforce due to the global restrictions,^68^ thereby worsening the already reduced work productivity in those with FH and losing a source of income essential for consistent management. Moreover, people with low‐income status were mostly affected by this displacement from work as their jobs were often not possible for remote services hence reducing their capability to afford necessary medications, diet plans and increased the chances of morbidity.^69^ Researchers have hypothesized that the indirect impacts of the COVID‐19 pandemic in low‐socioeconomic families is due to the inability to recoup lost wages from unemployment, as lower SES were more likely to be impacted by layoffs and the potential loss of employer‐provided health insurance.^70^, ^71^ Ultimately, the lack of general coverage prevails in low‐income households and will only worsen the care of FH due to the inability to meet the financial demands of management during the insecurities perpetuated by the pandemic.^72^

### 5.3 Racial and ethnic minorities disproportionately affected by COVID‐19

Over 90% of those affected by FH remain undiagnosed in underrepresented demographics and experience higher frequency of FH due to the founder effect.^73^ The lack of genetic testing, family screening, and prompt lipid‐lowering regimens are all factors that influence access to adequate healthcare measures, increasing the gap in health burden. Data from a large international initiative demonstrated how FH management and diagnosis can differ depending on demographics.^74^, ^75^ FH often remains under‐diagnosed, leading to under‐treatment and worse prognosis for patients. From this analysis, it is apparent that Western European countries present with a number of hospitals and clinics that are involved with FH research and management, whereas this seems not to be the case for a number of developing countries. FH treatment options, particularly PCSK9i, are not always easily and freely available to all, further impairing positive outcomes in LDL reduction for these patients.^75^

Adding onto disparities in adequate access to healthcare, it is known that non‐White individuals are highly affected by CVD. For instance, African Americans have been reporting higher incidence of CHD and Black men of any age are more likely to suffer from myocardial infarction compared to those of European ancestry (EA).^76^ Furthermore, compared to EA adults, Black men and women have been shown to suffer from ASCVD more commonly.^77^ A further concerning statistic, which is also relevant to FH, is that non‐White individuals are less likely to achieve ideal CV health parameters, putting this group of patients at an increased risk of CV risk factors, including hypertension and other parameters.^78^

Several hypotheses have been postulated as to why such differences exist. Social determinants of health are thought to be at the core of the difference in risk factors and outcomes of CVD experienced by non‐White patients suffering from FH. Some of the factors often described in the literature are issues in access to education, healthy food, stable living situations, all of which contribute to worse prognosis overall. For instance, it has been shown that African Americans and Latinos are less likely to comply with statins regimen following cardiac revascularization, something also correlated to the aforementioned social factors.^79^ Further analyses have also supported such findings; for instance, Agarwala et al. have demonstrated significantly lower use of statins and lipid‐lowering agents in Black patients suffering from FH, which they related to differences in access to care mostly due to psychological and/or social factors.^78^ Finally, the Cascade Screening for Awareness and Detection of FH (CASCADE‐FH) register has shown that non‐White patients with FH are half as likely to achieve optimal cholesterol control compared to EA patients with FH.^80^

Given the known connection between FH and COVID‐19 infection, where one can lead to exacerbations of the other and vice versa, it follows that non‐White patients are at particular risk of suffering from COVID‐19 complications in an indirect manner.^81^ Studies in Latin America, found that disadvantaged communities were more likely to suffer from COVID‐19 mortality due to differential access to healthcare and the higher prevalence of cardiac risk factors that are relevant to the risk of developing CV complications.^82^, ^83^ Indeed, taking the United States as an example, data has demonstrated that African Americans experienced a lack of COVID‐19 testing compared to White individuals, even though they represented a large proportion of deaths.^84^ An additional factor reported is that a lower percentage of African Americans had access to health insurance, which makes this group less likely to receive appropriate access to medications and testing when needed.^83^ However, there is a lack of literature pertaining to whether racial and ethnic disparities observed in FH and the COVID‐19 pandemic were related to one another. It may be hypothesized that the risk of COVID‐19‐related mortalities disproportionately affect minorities due to systemic healthcare barriers, which could interplay with the disparities of FH screening and treatment. Therefore, further research should be dedicated toward researching significant barriers during the COVID‐19 pandemic that disrupted FH care to disproportionately affected population groups.

### 5.4 Community‐wide impacts of social‐distancing and lockdown measures on healthcare systems

As with other uncommon diseases, HoFH and HeFH patients require consistent medical care even in the midst of pandemics. As a result of the current COVID‐19 crisis, which included social isolation and nation‐wide lockdowns, physical activity levels have decreased significantly.^85^ Patients with FH are classified as a high‐risk patient group during the pandemic, which warranted the government to issue pandemic‐related statements to isolate and maintain social distancing.^10^ However, many life‐style modifications that could have alleviated ASCVD risk in FH patients were potentially hindered by staying at home, resulting in sedentary lifestyles and excessive screen time, decreasing calorie consumption. The direct impact of the COVID‐19 pandemic was reportedly seen with reduced physical activity and increasingly becoming more sedentary, which often posed serious health risks in the long‐term.^86^

Nutritional care and physical activity are two important, but underrepresented interventions that can reduce ASCVD risk in FH participants. While it is important to note that life‐long nonpharmacological modifications are not sufficient to achieve desired LDL levels in FH participants, healthy lifestyle habits have been moderately correlated with protection against risk factors for atherosclerosis development.^87^ Nonpharmacological approaches are often promoted starting in adolescence in conjunction with pharmacotherapies. For example, higher cardiorespiratory fitness was associated with a lower risk of CVD mortality in male FH patients, underscoring the importance of physical activity in primary prevention of CVD.^88^ Dietary recommendations have also been a core component of CVD prevention in FH patients, as consumption of food rich with saturated fatty acids can increase LDL‐C; these outcomes could worsen if patients exhibit insulin‐resistant phenotypes such as diabetes mellitus, hyperinsulinemia, obesity, and elevated glucose and triglyceride indexes.^89^ Previous studies have denoted that better lifestyle interventions including healthy diet, exercise, not smoking, and maintaining a healthy weight were all significantly associated with reduced ASCVD in FH patients.^90^, ^91^ Those with FH genetic mutations also had similar positive results, indicating that such lifestyle interventions had a protective effect overall for patients suffering from different forms of the disease.^90^, ^91^ Similar benefits are also experienced by the general population when incorporating better lifestyle choices. It should, however, be noted that such interventions should be tailored to patients in the right way, otherwise they may risk being nonsignificant, as shows in a comparatively much smaller trial.^92^ In addition, dietary modifications should be considered on a case‐by‐case basis given the lack of international consensus as to whether low carbohydrate diets could significantly reduce atherosclerotic risk.^89^

The literature has not yet evaluated whether the COVID‐19 pandemic played a role in the shift away from healthy lifestyle modifications in FH participants due to social distancing measures and physical isolation. However, it was observed that these restrictions significantly impacted residents' lives irrespective of chronic disorders, particularly on their eating patterns and day‐to‐day behaviors. Two main factors that contributed to such outcomes included stockpiling food owing to the restrictions on grocery shopping and remaining at home (which includes digital learning, smart working, and limiting outdoor and indoor physical activity).^93^ In turn, the significant disruption to daily activity, coupled with the constant exposure to COVID‐19‐related news, caused participants to become increasingly stressed. This caused participants to become more prone to over‐eating sugary “comfort foods,” which are mostly high in simple carbs and are known to help lower stress by promoting serotonin synthesis.^94^, ^95^, ^96^ Beyond a chronic state of inflammation, which has been shown to raise the risk for more severe complications of COVID‐19, the effect of carbohydrates on food cravings is proportional to the glycemic index of foods, which is associated with an increased risk of developing obesity and CVD.^97^, ^98^, ^99^ However, it is unclear whether these factors highly influenced CV risk in FH and require more detail in future research investigations.

## 6 CURRENT PROSPECTS AND CHALLENGES IN CV TREATMENT AND PREVENTION FOR FAMILIAL HYPERCHOLESTEROLEMIA

FH puts individuals at an increased risk of CVD and early mortality, both of which are also factors associated with COVID‐19.^100^ In a bidirectional connection, COVID‐19 has also added further strains to healthcare systems across the world, in that it has lowered day to day care provision for patients, impairing prompt access to treatments and preventative measures.^101^ Since COVID‐19 was shown to also lead to endothelial deterioration and CVD occurrence, it follows that FH patients should receive adequate support and monitoring.

Primary prevention for FH would include pharmacological interventions and life‐style modifications. Currently, there is no final consensus regards to the optimal treatment protocol for FH, which varies depending on the country and local guidelines.^75^, ^102^ However, the main, and first‐line option for FH remains to be statins.^103^ Indeed, children with known heterozygous FH should start statin therapy by the age of 12. In children with homozygous FH, therapy should be started even more promptly, since complications tend to present earlier.^104^ In the case of COVID‐19, statin therapy serves a double role, that of lowering cholesterol levels, and that of lowering cytokine levels, thus reducing the high levels of inflammation caused by COVID‐19, largely central in the virus mortality.^105^, ^106^, ^107^ However, statins may often not work for a proportion of patients if desirable LDL‐C reduction was not achieved or due to adverse allergic reactions against these agents. As a result, a further treatment option for FH patients includes PCSK9 inhibitors, which have been proven to be superior compared to other lipid‐lowering agents such as resins and ezetimibe.^108^ These act to reduce possible CVD events by lowering atherosclerotic plaques. Interestingly, it was shown that PCSK9 inhibitors also have an anti‐inflammatory action, also leading to lowered cytokines levels, and clinically with reduced intubation needs and mortality in affected patients.^109^ Life‐style changes are also considered to improve long‐term ASCVD management in FH and are included in various clinical practice guidelines. For instance, the American Heart Association recommends the incorporation of a healthy diet alongside pharmacological management, which includes a higher intake of vegetables and fruit, low fat poultry and dairy products, as well as limited red meats and sweets.^110^ Caloric intake should also be adjusted to ensure patients avoid weight gain, and 3−4 sessions of physical exercise should be added per week. Similarly, the ESC, the European Atherosclerosis Society, and the Norwegian Advisory Unit for FH have all implemented a joint statement recommending the same guidelines. While lifestyle modifications may not be sufficient to achieve low enough levels of LDL‐C, the growing body of evidence regarding nongenetic modifiers suggests that a strong adherence to healthy lifestyle habits can reduce the risk of CAD irrespective of carrier status.^91^, ^111^ As a result, supporting FH patients with individually tailored lifestyle interventions may be key in facilitating these healthy changes and cocurrently improve adherence to statin therapy.^112^, ^113^

In addition to primary prevention strategies of ASCVD in FH, secondary prevention often relies on screening protocols and requires consistent monitoring throughout life. For example, available risk stratification protocols can be performed with Montreal‐FH‐SCORE^114^, ^115^ or SAFEHEART risk‐equation,^116^ which both have been shown to predict major CV events and mortality. However, the underdiagnosis of FH patients remains a public health issue that has yet to be addressed. Cascade screening was introduced as an evidence‐based intervention that significantly improved the uptake of FH diagnosis. The process started once an index patient meeting the diagnostic criteria for FH was identified, which thereafter initiated systemic family tracing starting with first‐degree relatives. Previous studies have demonstrated that screening could incorporate both molecular diagnoses and/or cholesterol tests, especially to cover functional mutations present within families, and reduce ASCVD morbidity. However, barriers to cascade screening occur when an index patient is not identified due to a lack of healthcare resources available for lipid testing and cholesterol screening within communities. Social disparities and access to facilities during the pandemic could significantly impact the utilization of cascade screening, especially with the lack of infrastructure that could facilitate testing from the level of primary care to refer to index patients. For example, Latin‐American countries such as Brazil observed a significant deterioration in FH services due to the closure of new admissions, reductions in routine lipid consultations, and a complete stop to the FH cascade screening program in the area.^117^ Outside of FH, the impact of the pandemic was visibly observed in the screening system for other conditions such as the hepatitis C cascade care program and nation‐wide cancer screening.^118^ Opportunistic testing is an additional method of screening offered to FH patients, and relies on a degree of clinical suspicion in general practice during routine health check‐ups. The recognition of FH in primary care depends on the capacity of community laboratories or healthcare worker‐based screening, but is limited in generalizability of the population due to the lack of systematic screening in these approaches. Similar to the issues raised with cascade screening, the shift in capacity for COVID‐related laboratory testing may have negatively impacted opportunistic screening approaches during the lockdown. Unfortunately, there are a lack of national studies that characterizes the impact of the pandemic on these screening protocols and its association with increased adverse coronary events in FH cohorts.

The prevention of ACSVD in FH often relies on the need to establish universal screening protocols and requires consistent monitoring throughout life. Unfortunately, there is a lack of studies that demonstrate whether the multifaceted effect of the pandemic, such as avoidance of medical care and reduction in consultations, significantly impacted wide‐scale screening for lipid levels in specific FH cohorts. In a current report, it was shown that a great percentage of FH patients were unable to carry their lifestyle regimen properly, experiencing disrupted sleep, changes in appetite, and increased anxiety.^56^ This can be coupled with the fear of contracting the virus, as well as having general difficulties adjusting to the new paradigm of healthcare, which can severely impair the prevention of cholesterol accumulation in FH patients. Overall, although COVID‐19 imposes a serious threat to FH patients, it has also not been considered in current FH treatment guidelines, which could be due to long update processes and a lack of research disseminating its outcomes.^119^ The resurgence of ASCVD and hereditary dyslipidemia post‐pandemic could potentially overwhelm healthcare resources and the above‐mentioned limitations, posing as a major public health concern in FH care.^120^ As a result, primary care, including preventive measures, and screening must be strengthened.

## 7 FUTURE RECOMMENDATIONS IN THE POST‐COVID 19 ERA

**Figure 3:**
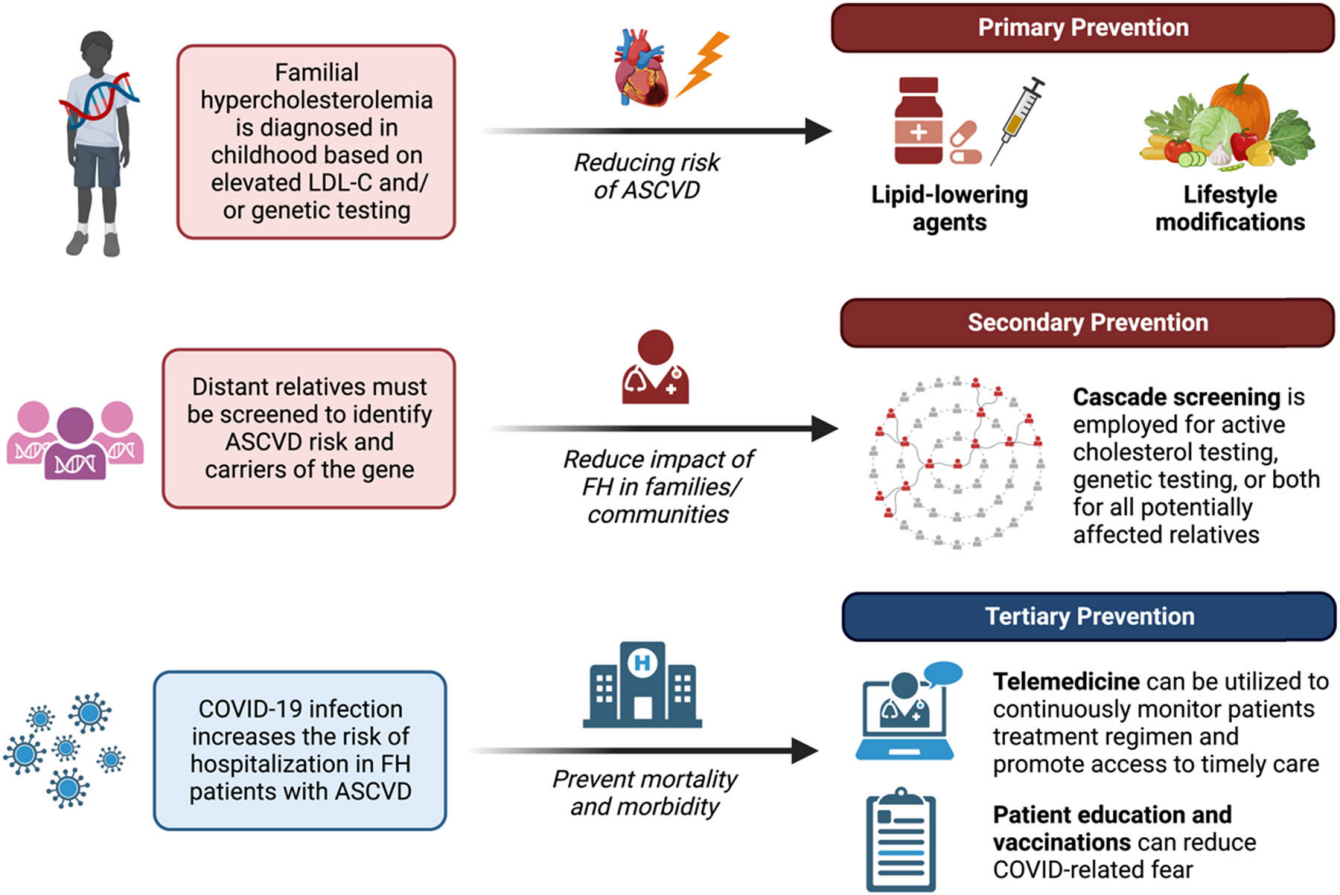
Levels of prevention for familial hypercholesterolemia and tertiary strategies applicable to the pandemic. ASCVD, atherosclerotic cardiovascular disease; LDL‐C, low‐density lipoprotein cholesterol.

There is a continued concern for the financial burden amongst other aspects of the healthcare system to address post‐COVID‐19 lockdown health‐related issues and unmet healthcare needs of patients. Worse still, the existing healthcare gaps in socioeconomic and financial status have further prevented FH patients from getting quality healthcare. Hence, it is vital to strengthen secondary and tertiary preventative measures, such as strengthening education and vaccinations to better inform the public and patients and equip FH patients with adequate knowledge to overcome the fear of seeking treatment^121^ (Figure 3). With the advancement of digital technologies, telemedicine is a feasible solution to overcome inefficient screening and narrow socioeconomic disparities to encourage and promote quality healthcare among patients, even from home.^122^ Given that FH patients require consistent care under specialists, utilization of telehealth can encourage higher referrals of FH screening, improves access to care in underserved communities, and better monitor FH patients with ASCVD. For example, a fourfold increase in utilization of telemedicine in the United States increased the uptake of posttreatment clinical encounters on HCV cascade care.^118^ Remote consultations can also be the solution in mitigating COVID‐related anxiety and is convenient, as low SES families are more likely to maintain multiple work obligations to meet financial ends.^123^, ^124^ Incorporating telemedicine into practice can help maximize risk factor control, track physical activity levels, keep up with patients' follow‐up and monitor diet while isolated. Though e‐health illiteracy is a common argument against telehealth usage, the implementation of telehealth garnered positive outcomes for patients, suggesting its feasibility in population‐wide studies.^125^

The disruption of screening programs during the pandemic can leave a significant impact on health‐related outcomes and requires a strong surge in healthcare capacity to catch‐up for missed screens. Therefore, the need to identify undiagnosed FH cases is crucial on a nation‐wide level by adopting universal screening and restructuring existing protocols. Cascade screening has been the most cost‐effective approach that has been implemented in multiple guidelines, but is often lackluster with its effectiveness to detect a larger number of FH families. Based on a study of the European Union, the availability of universal screening for FH is extremely limited compared to availability of cascade screening and traditional cholesterol testing.^126^ Yet, it was suggested that universal screening combined with cascade and opportunistic screening could significantly improve the rate of identification and early treatment. In Slovenia, the universal 3‐step FH screening approach amongst children was successful in reaching >91% of the pediatric population and detecting positive genetic variants in first‐degree relatives with high cholesterol with a cost‐effective analysis at around $900 for every screened case.^127^, ^128^, ^129^ These outcomes came after a retrospective analysis from 2012 to 2016 found that FH was confirmed in almost half of referred children through universal screening at primary care. Other countries with low‐prevalence of obesity such as Japan, have also found that universal screening of LDL‐C effectively identifies high‐risk patients with FH. Universal screening of children for FH at time of immunization was also proven cost‐effective in Western Australia, a region that experienced low detection of FH within at‐risk populations through solely relying on cascade screening, and deemed acceptable to the general public.^130^, ^131^

The support toward the effectiveness of incorporating universal screening strategies during routine primary care visits strongly suggests the importance of adopting universal protocols across countries.^132^ However, there continues to be a push‐back on universal screening protocols due to insufficient studies disseminating the benefits of universal screening on CV outcomes in FH and the potential costs from testing.^133^ In such cases, it is therefore important to shift the attention toward existing programs by adopting cost and value recommendations by increasing screening efforts to reduce morbidity, mortality, and the burden of disease. Promoting and implementing a “case‐finding strategy” approach to cascade screening poses to be a cost‐effective measure in counteracting clinical manifestations and complications of FH.^134^, ^135^ Moreover, improving uptake in cascade screening could be done by implementing the Dutch model to engage the FH Foundation with probands and relatives outside of healthcare settings and utilizing web‐based tools to increase communication between at‐risk relatives and FH probands.^134^, ^136^ Raising awareness of FH through public health strategies and educational initiatives may also increase pediatric lipid testing, but its effectiveness must continue to be explored.^137^ Genetic screening should also be emphasized to identify FH patients for early treatment. Given the rise of studies showing that COVID‐19 effects are much worse in FH patients, it may be helpful to employ this type of screening in CV prevention. Previous findings suggested that the there is a poor overlap in genetic etiology for FH and clinically diagnosed phenotypes, which further extend the importance of genetic testing due to heterogeneity.^138^ Genetic testing may refine risk estimates and could advance risk stratification based on causal gene variants.^139^ However, given the lack of literature characterizing the direct economic and racial disparities of COVID‐19 in FH, it may be difficult to identify direct solutions that can mediate the financial burden on patients and healthcare systems as a result of the pandemic. The potential challenges in genetic testing for FH patients such as cost and consensus on clinical guidelines is worth exploring and encourages the adoption of newer screening models considering the after‐effects of the pandemic.^139^, ^140^

Future research should be conducted to utilize resources that could ensure patients with FH are under regular care and constant therapy of statins to limit hospitalizations and improve their overall health outcomes. Further evidence based multicentre studies should consider establishing epidemiological trends in FH access amongst low‐SES communities and racial inequities to determine its relationship with CV outcomes and hospitalization. Insightful information on these aspects can help strategize health policies within governmental stakeholders while creating more efficient allocation of healthcare resources to address these needs.

## 8 CONCLUSION

Exploring the direct and indirect impact of the COVID‐19 pandemic on the CV outcomes and management of FH patients can strengthen current guidelines and advance novel recommendations. Due to the inheritance of genetic defects distinctive of FH, morbidity related to CV events among FH patients begins from a young age, especially among heterozygous individuals. However, the pandemic made patients with FH who also have ASCVD more likely to experience increased risk of COVID‐19 infection and thus face severe consequences. Fear of seeking out care during the pandemic, financial insecurity, socioeconomic and racial disparities, and the lack of physical activity during lockdown is perpetuated by the COVID‐19 pandemic and disproportionately affects CV outcomes in FH patients. Therefore, more resources must be fueled into early identification, diagnosis, management, and prevention of FH patients to reduce long‐term outcomes. The continued use of lipid‐lowering therapies such as statins is needed during the COVID pandemic and is recommended in long‐term prevention after recovery of COVID‐19 infection. However, future research can shape better recommendations surrounding the usage of telemedicine, genetic screening, and cascade screening.

## AUTHOR CONTRIBUTIONS

*Conceptualization of topic and coordination of reading, writing, and editing*: Helen Huang. R*eading, writing, and editing of the original draft*: Helen Huang, Keith S. K. Leung, Tulika Garg, Adele Mazzoleni, Goshen D. Miteu, Wireko A. Awuah, Elaine T. S. Yin, Faaraea Haroon, Zarish Hussain, and Narjiss Aji. *Critical revision of the manuscript*: Helen Huang, Keith S. K. Leung, Tulika Garg, Adele Mazzoleni, Goshen D. Miteu, Wireko A. Awuah, Vikash Jaiswal, and Gary Tse. *Figures and tables*: Helen Huang. *Final approval of manuscript*: Helen Huang, Keith S. K. Leung, Vikash Jaiswal, and Gary Tse.

## CONFLICT OF INTEREST STATEMENT

The authors declare no conflict of interest.

## Supporting information

Supporting information.

## Data Availability

No data is available.
