# Supplementary material for: Barriers and shortcomings in access to cardiovascular management and prevention for familial hypercholesterolemia during the COVID‐19 pandemic
Source: Clin Cardiol. 2023 Jun 1;46(8):831–44. doi: 10.1002/clc.24059 (PMC10436799; doi:10.1002/clc.24059)
Supplement: Supplementary file 1 — Supporting information. [file CLC-46-831-s001.docx]

**Supplementary Material**

| **Supplementary Table 1: Dutch Lipid Clinic Network (DLCN) Criteria** | |
| --- | --- |
| **Criteria** | **Score** |
| **Family history** | |
| Premature CVD (men <55 y old, women <60 y old) in first‐degree relative, OR | 1 |
| LDL >95th percentile in first‐degree relative AND/OR | 1 |
| Tendon xanthoma and/or corneal arcus in first‐degree relative, OR | 2 |
| LDL >95th percentile in children <18 y old | 2 |
| **Personal history** | |
| Premature CAD in patient (men <55 y old, women <60 y old) | 2 |
| Premature cerebral or peripheral vascular disease (men <55 y old, women <60 y old) | 1 |
| Clinical examination | |
| Tendon xanthomas, OR | 6 |
| Corneal arcus younger than 45 y old | 4 |
| **LDL** | |
| >330 mg/dL (8.5 mmol/L) | 8 |
| 250–329 mg/dL (6.5–8.5 mmol/L) | 5 |
| 190–249 mg/dL (4.9–6.4 mmol/L) | 3 |
| 155–189 mg/dL (4.0–4.9 mmol/L) | 1 |
| Presence of functional LDL‐R mutation (in the LDL‐R, ApoB, or PCSK9 gene) | 8 |
| **Diagnosis based on the overall score** | |
| Definite | >8 |
| Probable | 6–8 |
| Possible | 3–5 |
| Unlikely | <3 |

* Reproduced from McGowan MP, Hosseini Dehkordi SH, Moriarty PM, Duell PB. Diagnosis and Treatment of Heterozygous Familial Hypercholesterolemia. J Am Heart Assoc. 2019;8(24):e013225.

| **Supplementary Table 2: Simon Broome Criteria** | |
| --- | --- |
| **Criteria** | **Possibility** |
| In adults: TC >7.5 mmol/L (290.0 mg/dL) (or when available, LDL‐C >4.9 mmol/L [189.5 mg.dL])  In pediatric patients: TC >6.7 mmol/L (259.1 mg/dL), or LDL‐C >4 mmol/L (154.7 mg/dL), AND | Definite |
| Tendon xanthoma in the patient or first/second‐degree relative, OR alternatively: |  |
| Presence of LDL‐R, ApoB, or PCSK9 mutation |  |
| In adults: TC >7.5 mmol/L (290.0 mg/dL) (or when available, LDL‐C >4.9 mmol/L [189.5 mg.dL])  In pediatric patients: TC >6.7 mmol/L (259.1 mg/dL), or LDL‐C >4 mmol/L (154.7 mg/dL), AND | Possible |
| Family history of MI <50 y old in second‐degree relative or <60 y old in first‐degree relative OR alternatively |  |
| Family history of TC >7.5 mmol/L (290.0 mg/dL) in a first‐ or second‐degree relative. |  |

* Reproduced from McGowan MP, Hosseini Dehkordi SH, Moriarty PM, Duell PB. Diagnosis and Treatment of Heterozygous Familial Hypercholesterolemia. J Am Heart Assoc. 2019;8(24):e013225.

| **Supplementary Table 3: US MEDPED Criteria** | | | | |
| --- | --- | --- | --- | --- |
| **FH is diagnosed if total cholesterol exceeds the following cut‐off values in mg/dL (mmol/L)** | | | | |
| **Age (y/o)** | **First‐Degree Relative With FH** | **Second‐Degree Relative With FH** | **Third‐Degree Relative With FH** | **General Population** |
| <20 | 220 mg/dL (5.7 mmol/L) | 230 mg/dL (5.9 mmol/L) | 240 mg/dL (6.2 mmol/L) | 270 mg/dL (7.0 mmol/L) |
| 20–29 | 240 mg/dL (6.2 mmol/L) | 250 mg/dL (6.5 mmol/L) | 260 mg/dL (6.7 mmol/L) | 290 mg/dL (7.5 mmol/L) |
| 30–39 | 270 mg/dL (7.0 mmol/L) | 280 mg/dL (7.2 mmol/L) | 290 mg/dL (7.5 mmol/L) | 340 mg/dL (8.8 mmol/L) |
| ≥40 | 290 mg/dL (7.5 mmol/L) | 300 mg/dL (7.8 mmol/L) | 310 mg/dL (8.0 mmol/L) | 360 mg/dL (9.3 mmol/L) |

* Reproduced from McGowan MP, Hosseini Dehkordi SH, Moriarty PM, Duell PB. Diagnosis and Treatment of Heterozygous Familial Hypercholesterolemia. J Am Heart Assoc. 2019;8(24):e013225.

| **Supplementary Table 4: NLA Considerations** | |
| --- | --- |
| **Children, Adolescents, Young Adults < 20 years-old** | **Adults ≥ 20years-old** |
| LDL‐C ≥160 mg/dL (4.1 mmol/L)  Non‐HDL‐C ≥190 mg/dL (4.9 mmol/L) | LDL‐C ≥190 md/dL (4.9 mmol/L)  Non‐HDL ≥220 mg/dL (5.7 mmol/L) |
| At the LDL‐C levels listed below, the probability of FH is ≈80% in the setting of general population screening. These LDL‐C levels should prompt the clinician to strongly consider a diagnosis of FH and obtain further family information: | |
| LDL‐C ≥250 mg/dL (6.5 mmol/L) in a patient aged ≥30 y/o | |
| LDL‐C >220 mg/dL (5.7 mmol/L) for patients aged 20 to 29 y/o | |
| LDL‐C ≥190 mg/dL (4.9 mmol/L) in patients aged <20 y/o | |

* Reproduced from McGowan MP, Hosseini Dehkordi SH, Moriarty PM, Duell PB. Diagnosis and Treatment of Heterozygous Familial Hypercholesterolemia. J Am Heart Assoc. 2019;8(24):e013225.

| **Supplementary Table 5: Japan Atherosclerosis Society FH Criteria** |
| --- |
| **Primary diagnostic criteria for heterozygous FH in Adults (15 years of age or older)** |
| 1. Hyper-LDL-cholesterolemia (an untreated LDL-C level ≥ 180 mg/dL) |
| 1. Tendon xanthomas (thickening of tendons on dorsal side of the hands, elbows, knees or Achilles tendon hypertrophy) or xanthoma tuberosum |
| 1. Family history of FH or premature CAD (within the patient's second-degree relatives) |
| **Considerations to be taken:** |
| The diagnosis should be made after excluding secondary dyslipidemia |
| If a patient meets two or more of the above-mentioned criteria, the condition should be diagnosed as FH. In case of suspected heterozygous FH, making a diagnosis using genetic testing is desirable |
| Xanthelasma is not included in xanthoma tuberosum |
| Achilles tendon hypertrophy is diagnosed if the Achilles tendon thickness is ≥ 9 mm on X-ray imaging |
| An LDL-C level of ≥ 250 mg/dL strongly suggests FH |
| If a patient is already receiving drug therapy, the lipid level before treatment should be used as the reference for diagnosis |
| Premature CAD is defined as the occurrence of CAD in men < 55 years of age or women < 65 years of age, respectively |
| If FH is diagnosed, it is preferable to also examine the patient's family members |
| These diagnostic criteria also apply to HoFH |

* Reproduced from Harada-Shiba M, Arai H, Ishigaki Y, et al. Guidelines for Diagnosis and Treatment of Familial Hypercholesterolemia 2017. J Atheroscler Thromb. 2018;25(8):751-770.

| **Supplementary Table 6. Wales FH service genotype scoring criteria** | | |
| --- | --- | --- |
| **Family history** | 1^st^/2^nd^ degree relative: | |
|  | Known with premature (<60yrs) CHD | 1 |
|  | Known with premature (<45yrs) CHD | 2 |
|  | Known with LDL-C > 4.9 mmol/l (or total chol > 7.5 mmol/l) | 1 |
|  | <18 yrs with LDL-C > 4.0 mmol/l (or total chol > 6.7 mmol/l) | 2 |
|  | Please specify relation to index case | |
| **Physical examination** | Tendon xanthomata (in patient or 1st/2nd degree relative) | 6 |
|  | Premature corneal arcus (no score for arcus senilis) | 4 |
| **Clinical history** | Patient with premature CHD (<45 yrs) | 4 |
|  | Patient with premature CHD (<50 yrs) | 3 |
|  | Patient with premature CHD (<60 yrs) | 2 |
|  | Patient with premature (<60yrs) strokes and/or peripheral vascular disease | 1 |
| **Untreated or corrected LDL-C Concentrations (mmol/l)** | LDL-C ≥ 8.5 | 8 |
|  | LDL-C 6.5–8.4 | 5 |
|  | LDL-C 5.0–6.4 | 3 |
|  | LDL-C 4.0–4.9 | 1 |
|  | If untreated LDL- C values are unobtainable see attached sheet (Correction Factor Table) and calculate estimated value. | |
| **Fasting triglycerides (mmol/l)** | Triglyceride 2.5–3.4 | Minus 2 |
|  | Triglyceride 3.5–4.9 | Minus 3 |
|  | Triglyceride ≥ 5.0 | Minus 4 |
|  | Record in the narrative box any 2° causes that predispose to raised triglycerides, e.g. diabetes | |
| The highest score is circled from each section and the overall score is obtained by totalling all scores together. If the score is 6 or greater, the patient would be offered genetic testing for FH. If the score was below 6, the criteria form would need to be approved by the FH medical advisor before the patient was offered testing. | | |

* Reproduced from Haralambos K, Whatley SD, Edwards R, et al. Clinical experience of scoring criteria for Familial Hypercholesterolaemia (FH) genetic testing in Wales. Atherosclerosis. 2015;240(1):190-196.

| **Supplementary Table 7: FAMilial hypercholesterolaemia Case Ascertainment identification Tool (FAMCAT)** |
| --- |
| Sex (male or female) |
| Age in years (16–24; 25–34; 35–44; 45–54; 55–64; 65–74; 75–84) |
| Highest cholesterol measurement recorded (mmol/l)  Ideal: TC ≤5 or LDL-C ≤3.3  High: TC >5 to ≤6.5 or LDL-C >3.3 to ≤4.1  Very high: TC >6.5 to ≤7.5 or LDL-C >4.1 to ≤4.9  Extremely high: TC >7.5 or LDL-C >4.9 |
| Triglycerides within 1 month of highest cholesterol measurement (mmol/l)  Idea: <1.7  Borderline high: ≥1.7 to <2.3  High: ≥2.3 to <5.6  Very high: ≥5.6  Not assessed |
| Lipid-lowering drugs prescribed within 1 month of highest cholesterol measurement (none; fibrate, bile acid sequestrant, or nicotinic acid; low-potency statin; medium-potency statin; high potency statin) |
| Family history of familial hypercholesterolaemia (no or yes) |
| Family history of myocardial infarction (no or yes) |
| Family history of raised cholesterol (no or yes) |
| Type 1 or type 2 diabetes (no or yes) |
| Chronic kidney disease (no or yes) |

* Reproduced from Akyea RK, Qureshi N, Kai J, et al. Evaluating a clinical tool (FAMCAT) for identifying familial hypercholesterolaemia in primary care: a retrospective cohort study. BJGP Open. 2020;4(5):bjgpopen20X101114.
